# MR1 deficiency enhances IL-17-mediated allergic contact dermatitis

**DOI:** 10.3389/fimmu.2023.1215478

**Published:** 2023-06-20

**Authors:** Naoya Imahashi, Masashi Satoh, Emanuela Clemente, Kazuhisa Yoshino, Mario Di Gioacchino, Kazuya Iwabuchi

**Affiliations:** ^1^ Program in Cellular Immunology, Graduate School of Medical Sciences, Kitasato University, Sagamihara, Japan; ^2^ Department of Immunology, School of Medicine, Kitasato University, Sagamihara, Japan; ^3^ Center for Advanced Studies and Technology (CAST), G. d’Annunzio University of Chieti-Pescara, Chiete, Italy; ^4^ Department of Anesthesiology, School of Medicine, Kitasato University, Sagamihara, Japan; ^5^ Institute of Clinical Immunotherapy and Advanced Biological Treatments, Pescara, Italy

**Keywords:** innate T cells, delayed-type hypersensitivity, neutrophils, gamma/delta T cells, allergy

## Abstract

Major histocompatibility complex (MHC) class Ib molecules present antigens to subsets of T cells primarily involved in host defense against pathogenic microbes and influence the development of immune-mediated diseases. The MHC class Ib molecule MHC-related protein 1 (MR1) functions as a platform to select MR1-restricted T cells, including mucosal-associated invariant T (MAIT) cells in the thymus, and presents ligands to them in the periphery. MAIT cells constitute an innate-like T-cell subset that recognizes microbial vitamin B_2_ metabolites and plays a defensive role against microbes. In this study, we investigated the function of MR1 in allergic contact dermatitis (ACD) by examining wild-type (WT) and MR1-deficient (MR1^-/-^) mice in which ACD was induced with 2,4-dinitrofluorobenzene (DNFB). MR1^-/-^ mice exhibited exaggerated ACD lesions compared with WT mice. More neutrophils were recruited in the lesions in MR1^-/-^ mice than in WT mice. WT mice contained fewer MAIT cells in their skin lesions following elicitation with DNFB, and MR1^-/-^ mice lacking MAIT cells exhibited a significant increase in IL-17-producing αβ and γδ T cells in the skin. Collectively, MR1^-/-^ mice displayed exacerbated ACD from an early phase with an enhanced type 3 immune response, although the precise mechanism of this enhancement remains elusive.

## 1 Introduction

The skin is repeatedly exposed to various antigenic substances of natural origin, cosmetics, metal accessories, and medical products of artificial origins, in the broad context of the environment (1), and the immunogenicity of these substances as sensitizers has been investigated (2, 3). Antigen-presenting cells (APC) in skin exposed to sensitizers migrate to the draining lymph nodes (dLN) *via* lymphatic vessels and present them in the context of gene products of self-major histocompatibility complex (MHC) class Ia and II to antigen (Ag)-specific T cells (4). Thus, Ag-specific CD8^+^ and CD4^+^ T cells are primed, and these Ag-specific T cells within the memory fraction may be activated upon Ag re-exposure and migrate to the site of Ag entry to induce allergic contact dermatitis (ACD). ACD is transferable with T cells but not with antibodies and is thus classified as T cell-mediated type IV hypersensitivity according to the Gell and Coombs classification (4).

An experimental model of ACD is often employed in mice by painting chemicals such as dinitrohalobenzene onto the skin to study the sensitization and elicitation phases in detail (4). In addition to T cells, various other immune and non-immune cells in the skin are involved in the pathogenesis of ACD, and the crosstalk among them has been studied (5–7). Keratinocytes are the main type of non-immune cells in the skin, constituting a barrier layer since they not only form a physical barrier against the entry of foreign substances and pathogens but also secrete IL-1β when sensing insults against the skin to transmit signals downstream to immune cells (5). The cells of innate immunity include Langerhans cells, dermal dendritic cells, macrophages, neutrophils, and mast cells, which present Ag information and affect the intensity of ACD (6). Natural killer (NK) cells and innate lymphoid cells (ILCs), lymphocytes without rearranged Ag-specific receptors, potentiate (NK and ILC1 in particular) or regulate (ILC2 in particular) the immune and inflammatory responses at both the sensitization and elicitation phases of ACD depending on the context (6, 7).

Innate-like lymphocytes with rearranged TCRs are also important components in ACD. Murine skin is known to harbor a special γδ T-cell population referred to as dendritic epidermal T cells (DETCs) expressing invariant Vγ3Vδ1 (in Garman Nomenclature [GN], Vγ5Vδ1 in Heilig-Tonegawa Nomenclature [H-TN]) TCR in the epidermis (8). However, humans do not have an equivalent epidermal T-cell population, although they harbor Vδ1^+^ and Vδ2^+^ T cells in the epidermis and dermis (9). Murine DETCs express NKG2D, which recognizes stress molecules such as RAE-1 induced in keratinocytes when sensitizing chemicals are applied to the epidermis (10). Moreover, keratinocyte-derived IL-1β induces IL-17 expression by DETCs (11) and Vγ2^+^ (in GN, Vγ4 in H-TN) or Vγ4^+^ (in GN, Vγ6 in H−TN) γδ T cell subsets, including others (collectively referred to as Tγδ17 cells) in the dermis (9), where the latter appear to play a more important role in ACD.

The skin also harbors innate-type T cells with αβ-type TCRs, including natural killer T (NKT) cells (12), and mucosal-associated invariant T (MAIT) cells (13), whose reactivities are restricted by MHC class Ib molecules, cluster differentiation 1d (CD1d), and MHC-like protein 1 (MR1), respectively. These T-cell subsets are also categorized as preset T cells and resemble each other in several ways (14): 1) They recognize non-peptide antigens of microbial origin in the context of the restricting class Ib (glycolipids/CD1d vs. vitamin B_2, 9_ metabolite/MR1), 2) major subsets utilize invariant Vα chain (mouse Vα14Jα18/human Vα24Jα18 vs. mouse Vα19/Jα33/human Vα7.2Jα33) with limited yet diverse Vβ chains, respectively, 3) the invariant subsets of T cells exhibit effector/memory phenotypes and may function as either effector or regulatory cells in health and diseases (15). Studies of these invariant T cells may provide insights as to controlling ACD with low-molecular-weight ligands without concerns about MHC barriers because the restriction molecule is homogenous in an allogeneic relationship and highly conserved even in xenogeneic combinations (14).

The role of NKT cells in ACD has already been investigated by employing gene knockout (^-/-^) mice, CD1d^-/-^ (whole NKT cell-deficient), or Jα18^-/-^ (invariant NKT [iNKT] cell-deficient) mice compared with wild-type (WT) mice with a C57BL/6 or BALB/c background (16–18). Initial studies demonstrated that ear swelling was reduced in both CD1d^-/-^ and Jα18^-/-^ mice, suggesting that iNKT cells appear to function in the initiation and enhancement of ACD through prompt induction of IL-4 after Ag exposure, with involvement of IgM^+^ B-1 B cells and effector αβ T cells (16, 17). Subsequent studies revealed that the differential functions of iNKT cells were dependent on the contact sensitizers employed in each study, with iNKT cells playing either pathogenic or regulatory roles (18, 19). Human studies have also demonstrated that iNKT cells are detected in ACD lesions, implying some critical roles (20, 21).

The involvement of another innate αβ type T cell, MAIT cells, in ACD has been limited to date and has been reported for palladium allergy in the foot pad lesions of BALB/c mice, where MAIT TCR was detected with iNKT TCR and presumed to display Ag-specificity (22). The role of MAIT cell accumulation in the lesion remains elusive in the development of ACD as a player in either inflammatory or regulatory responses. Thus, in the present study, we examined the effect of MR1/MAIT deficiency on ACD by comparing DNFB-induced ACD in WT versus MR1^-/-^ mice to probe for altered responses in MR1^-/-^ mice. The involvement of other subsets of innate-like T cells was also revealed in MR1^-/-^ mice, and their relevance in ACD is discussed.

## 2 Materials and methods

### 2.1 Mice

C57BL/6 (B6) mice were purchased from CLEA Japan, Inc. (Tokyo, Japan) and B6.MR1^-/-^ mice were kindly provided by Dr. Susan Gilfillan (Department of Pathology and Immunology, Washington University School of Medicine, St. Louis, MO, USA) (23) and housed and maintained in an animal facility at the Analysis Center for Integral Genomic Functions at Kitasato University School of Medicine. The mice were provided food and water *ad libitum*. All animals were humanely treated and housed under pathogen-free conditions. All experimental procedures involving mice conformed to the guidelines of the Animal Experimentation and Ethics Committee of Kitasato University School of Medicine (#2017-143, 2018-119, 2019-025, and 2022-079).

### 2.2 Induction of allergic contact dermatitis with 2,4-dinitrofluorobenzene

Mice were sensitized at shaved abdomen sites with 25 μL of 0.5% 2,4-dinitrofluorobenzene (DNFB) (Sigma-Aldrich, MO, USA) dissolved in a 1:4 mixture of olive oil (Nacalai Tesque, Inc., Kyoto, Japan): acetone (Fujifilm Wako Pure Chemical Co. Ltd., Osaka, Japan), as previously described (24). Five days after sensitization, the right pinna was painted with 20 μL of 0.3% DNFB, and the left pinna was painted with 20 μL of vehicle alone (elicitation/challenge). Each pinna was measured with a digital micrometer (Mitutoyo Corp., Kawasaki, Japan), and the net pinna thickness (Δthickness = thickness of right pinna – thickness of left pinna) was calculated at 0 (before), 1, and 2 days after challenge.

### 2.3 Cell preparation from pinnae and lymph nodes of treated mice

Right and left pinnae and inguinal lymph nodes (draining lymph nodes [dLN]) on the right and left sides of the mice were obtained after euthanasia using a confirmed procedure. The pinnae were used for histology, flow cytometry, functional analyses of infiltrated cells, and gene expression analyses. A single-cell suspension was prepared according to the protocol previously described with slight modifications (25). In brief, the removed pinnae were cut into pieces and incubated with 100 μg/mL Liberase^®^ and 400 ng/mL DNase I (both from Roche Diagnostics, K.K., Tokyo, Japan) at 37°C with gentle shaking for 1 h. The digestion was stopped by adding ice-cold phosphate-buffered saline without Ca^2+^ and Mg^2+^ [PBS (-)], and the solution was layered on Lympholyte^®^-M medium (Cedarlane Laboratories Ltd., Ontario, Canada) followed by centrifugation at 1,800 × *g* for 20 min. Cells at the interface were collected, washed with medium, and used for flow cytometry and cell culture. The lymph nodes were gently dispersed using a frosted-glass homogenizer to obtain a single-cell suspension, which was used for flow cytometry and cell culture.

### 2.4 Flow cytometric analysis

A single-cell suspension prepared as above was incubated with TruStain FcX™ anti-mouse CD16/32 antibody (BioLegend, CA, USA) and stained with the following mAbs: anti-mouse Ly-6G (1A8), CD11b (M1/70), TCRβ (H57-597), CD3 (2C11), CD4 (GK1.5), γδ TCR (GL3), Vγ2 (in GN; UC3-10A6), B220 (RA3-6B2), IL-17A (TC11-18H10.1), IFN-γ (XMG1.2), and T-bet (4B10) purchased from BioLegend, anti-mouse CD45.2 (104), RORγt (Q31-378BD) purchased from BD Biosciences, and anti-mouse Foxp3 (FJK-16s) purchased from Invitrogen. 5-OP-RU loaded MR1 tetramer was provided by the National Institute of Health Tetramer Core Facility at Emory University (Atlanta, GA, USA). Cells positive for 7-amino actinomycin D (BioLegend) were electronically gated as dead cells and excluded from the analysis. For transcription factor staining, the cells were initially stained with surface markers, and then fixed and permeabilized with the True-Nuclear™ Transcription Factor Buffer Set (BioLegend). For intracellular cytokine staining, cells were stimulated with PMA (50 ng/mL, Sigma-Aldrich) and ionomycin (500 ng/mL, Sigma-Aldrich) for 4 h in the presence of brefeldin A (x1000; BioLegend) before cell surface staining. The samples were washed and filtered and then analyzed by FACS. After surface staining, the cells were fixed and permeabilized with Fixation Buffer (BioLegend) and Intracellular Staining Permeabilization Wash Buffer (BioLegend), followed by staining with anti-cytokine mAbs. The stained cells were subjected to flow cytometry (FACSVerse™, BD Biosciences) and analyzed using FlowJo software (FlowJo, LLC, CA, USA). The flow cytometry was performed as described previously (25).

### 2.5 Cell culture and stimulation with dinitrobenzene sulfonic acid

At day 5 of DNFB sensitization, dLN cells were harvested in RPMI-1640 medium (Sigma-Aldrich) containing 10% FCS, 50 μM β-mercaptoethanol (GIBCO, MA, USA), 100 units/mL penicillin and 100 μg/mL streptomycin (Sigma-Aldrich). One million (1 × 10^6^) cells were cultured in the presence of 100 μg/mL dinitrobenzene sulfonic acid (DNBS) (Sigma-Aldrich) for 3 days (24) and the supernatant was collected for cytokine measurement, as described in section 2.6.

### 2.6 Quantification of cytokines

The concentration of Th1/Th2/Th17 cytokines in the culture supernatant was quantified by flow cytometry using a BD CBA Mouse Th1/Th2/Th17 Cytokine Kit (BD Biosciences, CA, USA) according to the manufacturer’s protocol.

### 2.7 Quantitative real-time PCR

Total RNA was extracted using the TRIzol^®^ reagent (Thermo Fisher Scientific). cDNA was synthesized from the total RNA using PrimeScript™ RT Master Mix (TaKaRa Bio Inc., Kusatsu, Japan). Real-time PCR was performed using TB Green^®^ Premix Ex Taq™ II (TaKaRa Bio Inc.) and a CFX96 real-time PCR detection system (Bio-Rad, Hercules, CA, USA), according to the manufacturer’s protocol. The target gene expression was normalized to *β-actin* and calculated using the 2^−ΔΔCT^ method. The primers were as follows: *Actb* (forward 5′-GGCTGTATTCCCCTCCATCG-3′; reverse 5′-CCAGTTGGTAACAATGCCATGT-3′), *Cxcl1* (forward 5′-TTGAAGGTGTTGCCCTCAGG-3′; reverse 5′-CCAGACAGGTGCCATCAGAG-3′), *Cxcl2* (forward 5′-GGCGGTCAAAAAGTTTGCCT-3′; reverse 5′-CAGGTACGATCCAGGCTTCC-3′), *Csf3* (forward 5′-GTTCCCCTGGTCACTGTCAG-3′; reverse 5′-TGGCTTAGGCACTGTGTCTG-3′), *Il17* (forward 5′-TGAAGGCAGCAGCGATCA-3′; reverse 5′-GGAAGTCCTTGGCCTCAGTGT-3′), *Il1b* (forward 5′-GCAACTGTTCCTGAACTCAACT-3′; reverse 5′-ATCTTTTGGGGTCCGTCAACT-3′) (Hokkaido System Science, Sapporo, Japan).

### 2.8 Histology and quantitative analyses of microscopic images

The pinna tissue was fixed with buffered formaldehyde solution (10%) (Fujifilm-Wako Pure Chemical), followed by the standard protocol for paraffin-embedded sections and hematoxylin-eosin (HE) staining. Images of the HE-stained tissue were captured using a BIOREVO microscope (BZ-X800, KEYENCE Corp., Osaka, Japan), and the thickness of the pinna was quantified using image analysis software (BZ-X) for the microscope, in addition to manual measurement with a digital micrometer, as described in Section *2.2*.

### 2.9 Statistics

The results are presented as means ± standard deviation (s.d.). Statistical analysis between two groups was performed using the Mann–Whitney U test, and comparison among three groups was performed using ANOVA followed by Tukey–Kramer tests. Values with *p* < 0.05 were considered statistically significant.

## 3 Results

### 3.1 MR1^-/-^ mice develop augmented ACD

**Figure 1:**
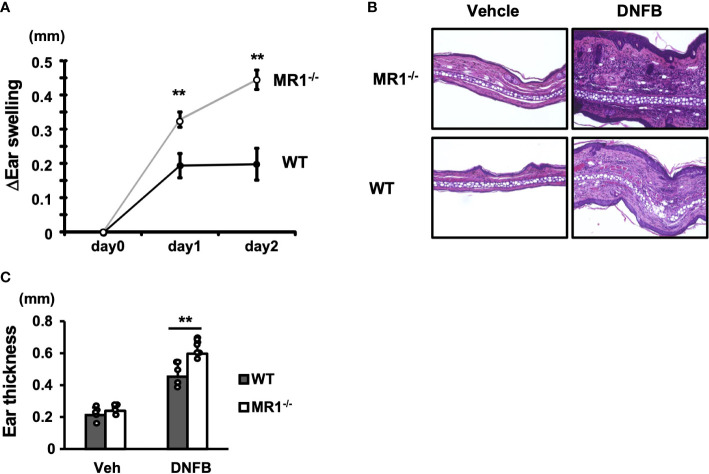
MR1^-/-^ mice develop augmented ACD compared with WT mice. **(A)** Wild-type (WT, C57BL/6; B6, closed circle) mice and B6.MR1^-/-^ (MR1^-/-^, open circle) mice were sensitized with 0.5% DNFB and challenged after five days on the left pinna with vehicle only or on the right pinna with 0.2% DNFB. The thickness of the pinnae was then measured with a digital micrometer on day 0 (day of challenge), day 1, and day 2. The increment in thickness of the sensitized pinna was presented as ΔEar swelling (mm), as described in the Materials and Methods. **(B)** Histology of vehicle-painted (control) and DNFB-painted (experimental) pinnae obtained from WT and MR1^-/-^ mice. **(C)** The net thickness of the pinnae including that of vehicle control deduced from the measurements of morphometric analyses of histological specimens is presented as Ear thickness (mm). Representative data of at least three experiments of four to five mice/experiment. Mann–Whitney *U* test. **p < 0.01.

To examine the role of MR1/MAIT cells in ACD, WT and MR1^-/-^ mice were sensitized with DNFB in an acetone/olive oil solvent on the abdominal skin and challenged five days later on the right pinna, and the increment in thickness of the pinna in each mouse was calculated. MR1^-/-^ mice exhibited a significantly greater increase in ear swelling than WT mice on days 1 and 2 after DNFB challenge (**Figure 1A**). MR1^-/-^ mice exhibited thicker pinnae, with severe intercellular edema and augmented infiltration of inflammatory cells compared to WT mice, as shown by histopathology (**Figure 1B**). The inflammatory cells in the DNFB-painted pinnae appeared to consist mainly of polymorphic neutrophils in both WT and MR1^-/-^ mice. The mean ear thickness of pinna painted with DNFB was also quantified using histological images, and that of MR1^-/-^ mice was greater than that in WT mice (**Figure 1C**). Although the representative histology of the vehicle control in MR1^-/-^ mice was slightly thicker than that in WT mice (**Figure 1B**), the mean ear thickness in the control group was similar between the WT and MR1^-/-^ mice.

### 3.2 More neutrophils are recruited into the ACD-induced pinna in MR1^-/-^ mice

**Figure 2:**
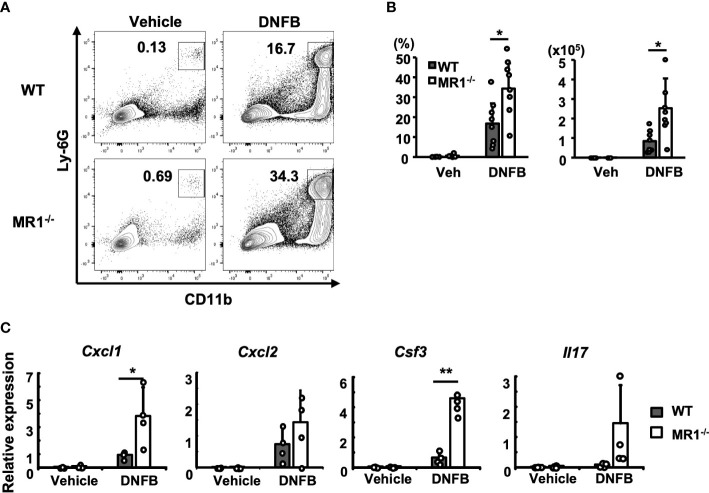
More neutrophils are recruited into the ACD-induced pinnae in MR1^-/-^ mice than WT mice. **(A)** Representative flow cytometric profiles of the cells infiltrated into the pinnae prepared two days after challenge with enzymatic degradation, as described in the Materials and Methods and analyzed according to the gating described for **Supplementary Figure 1A**. The square gate (CD11b^+^Ly6G^hi^ cells) indicates neutrophils. **(B)** Frequency of the CD45^+^ fraction and cell number in MR1^-^ **^/-^** mice compared with the fraction and cell number of WT mice represented by panel **(A)**. **(C)** The expression of *Cxcl1*, *Cxcl2*, *Csf3*, and *Il17* related with neutrophil recruitment was examined with mRNA obtained from the left pinnae (vehicle control) and the right pinnae (DNFB) two days after challenge. Representative data of at least three experiments of four to eight mice/experiment. Mann–Whitney *U* test. *p < 0.05, **p < 0.01.

To analyze inflammatory cells infiltrating the pinna challenged with control vehicle or DNFB, cells infiltrated into the pinna were obtained by disintegration of the tissue and analyzed by flow cytometry, as described in the *Materials and Methods*. The acquired mononuclear cells were gated as described (**Supplementary Figure 1A**). The neutrophils in the pinnae were identified as Ly6G^+^CD11b^+^ cells (**Figure 2A**). More neutrophils were recruited to the DNFB-challenged pinnae in both WT and MR1**^-/-^** mice (**Figure 2A**, right panels) than to the control pinnae (**Figure 2A**, left panels). Furthermore, the number and frequency of neutrophils in the challenged pinnae of MR1^-/-^ mice were significantly higher than those in the pinnae of WT mice (**Figure 2B**). The expression of genes related to neutrophil migration and survival, such as *Cxcl1, Cxcl2*, and *Csf3*, was significantly increased or tended to be increased in MR1^-/-^ mice compared to WT mice (**Figure 2C**). *Il17*, which stimulates the expression of these genes, also tended to be increased in MR1^-/-^ mice after two days of DNFB challenge (**Figure 2C**).

The Ly6G^lo-(-)^CD11b^+^ population that appeared straight below the neutrophil gate in **Figure 2A** was further separated into Ly6C^hi^F4/80^lo^ (monocyte/macrophage; Mo/Mϕ) and Ly6C^-^F4/80^+^ (macrophage; Mϕ) cells (**Supplementary Figure 2A**). Although tissue-resident Mϕ appeared to be the main cell type in the Ly6G^lo-(-)^CD11b^+^ population, Mo/Mϕ became dominant presumably *via* migration and Mϕ appeared to be markedly reduced by contrast (**Supplementary Figure 2A**, flow panels) and as a percentage (**Supplementary Figure 2B** graphs) during the elicitation phase.

### 3.3 Increased dermal γδ T cells in the pinnae of MR1^-/-^ mice

**Figure 3:**
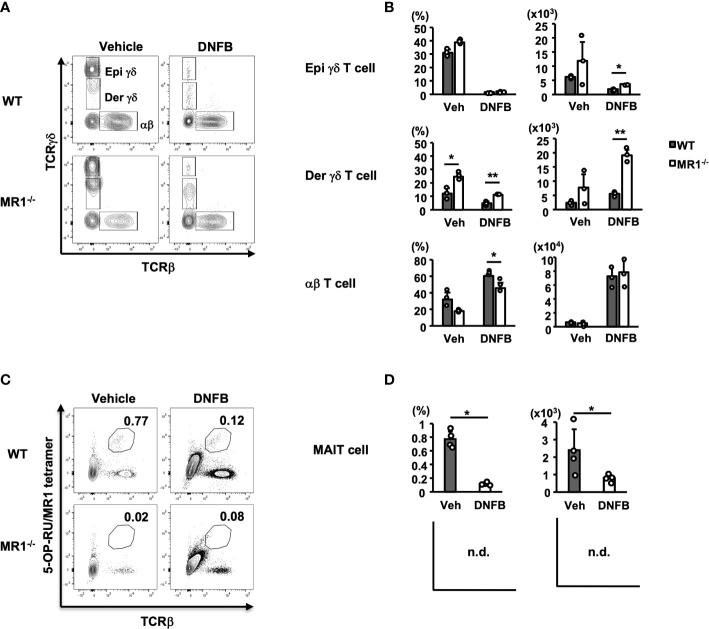
T-cell subsets in both vehicle control and DNFB-painted pinnae two days after challenge in WT and MR1^-/-^ mice. **(A)** Representative flow cytometric profiles of T-cell subsets in vehicle- and DNFB-painted pinnae. The cells were prepared as for **Figure 2** and analyzed according to the gating of **Supplementary Figure 1B** for γδ T and αβ T cells. The γδ^hi^ fraction is designated epidermal γδ T cells (Epi γδ) and the γδ^lo^ fraction as dermal γδ T cells (Der γδ). **(B)** Graphs of the frequencies and cell numbers for the αβ T, Epi γδ T, and Der γδ T cells represented in panel **(A)**. **(C)** Representative flow cytometric profiles of MAIT cells, analyzed with the gated fraction of lymphocyte CD45^+^ cells stained with 5-OP-RU/MR1-tetramer (kindly provided by NTCF, Atlanta, GA, USA) and anti-TCRβ mAb in vehicle control and DNFB-painted pinnae of WT mice two days after challenge. **(D)** Graphs of the frequencies and cell numbers for MAIT cells for the WT mice represented in panel **(C)**. MR1**^-/-^** mice lack MR1-restricted cells, including MAIT cells, graphs were not demonstrated (n.d.), with no difference between control and DNFB groups in trace amounts. Representative data of at least three experiments of three to four mice/experiment. Mann–Whitney *U* test. *p < 0.05, **p < 0.01.

To examine another major population of cells residing in control pinnae or infiltrating inflamed pinnae, we analyzed αβ- and γδ-type T cells of vehicle- and DNFB-treated pinnae by flow cytometry based on gating, as shown in **Supplementary Figure 1B**. Both αβ and γδ T cells were detected in vehicle control and DNFB-painted pinnae, and γδ T cells were clearly separated according to the fluorescence intensity as epidermal (Epi: TCR^hi^) and dermal (Der: TCR^lo^) γδ T cells (**Figure 3A**) (25). Notably, in the pinnae of the vehicle control group, the contour of αβ T cells was more evident in WT mice, whereas that of Der γδ T cells was more evident in MR1^-/-^ mice (**Figure 3A**, upper and lower left panels). Elicitation by DNFB caused a reduction (dense contour to scarce one or dots) of Epi and Der γδ T cells, whereas a clear population of αβ T cells was observed in both WT and MR1^-/-^ mice (**Figure 3A**, upper and lower right panels). When the number of T cells was analyzed further, the αβ T cells in the pinnae significantly increased after challenge with DNFB in both WT and MR1^-/-^ mice at similar levels, suggesting that the sensitized population of αβ T cells was vigorously recruited into the pinnae after painting in both strains of mice (**Figure 3B**, lower right panels), although the percentage of αβ T cells of MR1^-/-^ mice was significantly lower than that of WT mice due to the increased Der γδ T cells as described below (**Figure 3B**, lower left panels). Accordingly, the percentage of Epi γδ T cells was markedly decreased in the DNFB-challenged pinnae compared to the vehicle controls (**Figure 3B**, upper left panels). The number of Epi γδ T cells also appeared to be decreased in the DNFB-challenged group, whereas the extent was not as marked as that of the frequency, and MR1^-/-^ mice exhibited higher numbers than WT mice (**Figure 3B**, upper right panels). Another subset, Der γδ T cells, appeared to be decreased in frequency in WT and MR1^-/-^ mice in the DNFB-treated group under the influence of the dominant recruitment of αβ T cells, whereas the frequency was significantly higher in MR1^-/-^ mice than in WT mice in both the control and DNFB groups (**Figure 3B** middle left panel). Moreover, the number of Der γδ T cells was not reduced, even in WT mice, and was significantly increased in DNFB-challenged pinnae in MR1^-/-^ mice compared with WT mice (**Figure 3B** middle right panel). In contrast, the cells of interest in the present study, MAIT cells detected by 5-OP-RU/MR1 tetramer, were reduced in DNFB-challenged pinnae in comparison with those in the vehicle control, (**Figure 3C**) both in terms of frequency and cell number (**Figure 3D**). Since MR1**^-/-^** mice lack MAIT cells due to the *Mr1*-disruption, there was no difference between the control and DNFB groups.

The increment and reduction of each T cell subset, as compared with other immune cells among the different panels, are not evident, because the cell numbers during the pre- and post-elicitation stages of each cell number differ over several log scales. To better visualize the relationship of each subset of cells in the pinnae of vehicle- and DNFB-painted WT or MR1^-/-^ mice, the cumulative graph of cells for lymphocytic and phagocytic lineages is shown in **Supplementary Figure 3**. The majority of cells infiltrating the pinnae consisted of neutrophils, Mo/Mϕ, and αβ T cells in both WT and MR1^-/-^ mice, although there was a difference in the composition between WT and MR1^-/-^ mice in the vehicle control and DNFB-challenged pinnae.

### 3.4 Enhanced Th17 immune responses in MR1^-/-^ mice

**Figure 4:**
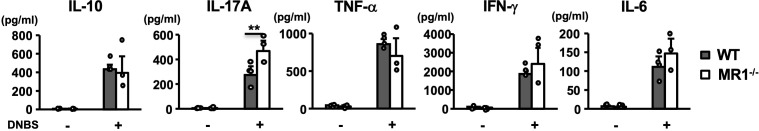
Cytokine production by antigen-specific T cells in draining lymph nodes. Lymph node T cells harvested from inguinal lymph nodes at day 5 in vehicle- and DNFB-painted WT and MR1^-/-^ mice were cultured for three days in the presence and absence of DNBS (100 μg/mL). Cytokines (IL-10, IL-17A, TNF-α, IFN-γ, IL-6) in the supernatant were quantified as described in the *Materials and Methods*. Representative data of two experiments of three to four mice/experiment. Mann–Whitney *U* test. **p < 0.01.

To examine the effect of MR1/MAIT cell deficiency on T-cell cytokine production in ACD, the whole draining LN (dLNs; inguinal) cells of abdominal skin from WT and MR1**^-/-^** mice were stimulated with 2,4-dinitrobenzene sulfonic acid (DNBS) *in vitro*. The level of IL-17A in the culture supernatant when stimulated with DNBS was significantly higher for the LN cells of MR1**^-/-^** mice than for those of WT mice (**Figure 4**). The production of other cytokines such as IL-10, TNF-α, IFN-γ, and IL-6 was comparable between WT and MR1**^-/-^** mice (**Figure 4**), and IL-4 production was almost undetectable (data not shown).

**Figure 5:**
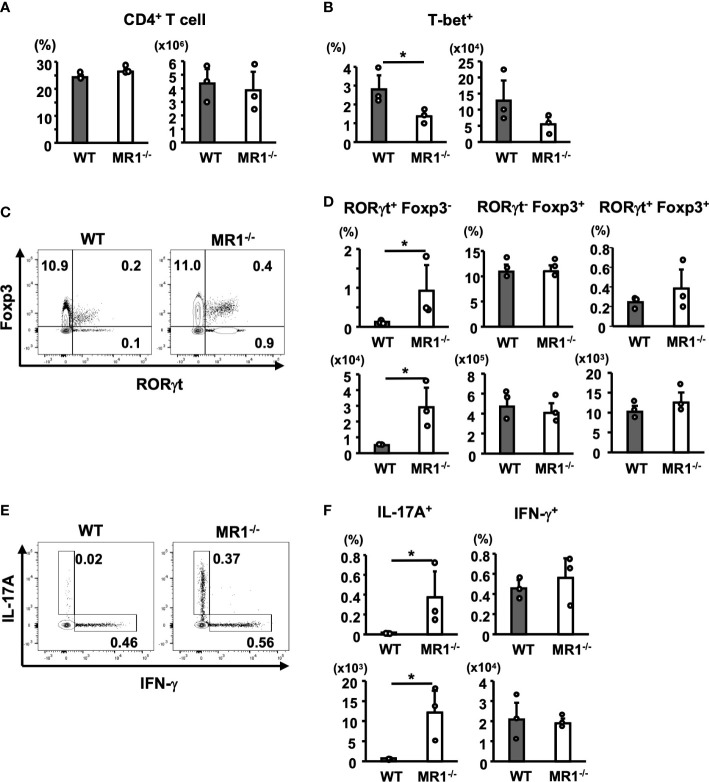
T-helper (Th) cell subsets in draining lymph nodes from WT and MR1^-/-^ mice. Cells in inguinal lymph nodes after five days of sensitization were obtained and stained for analyses described in the Materials and Methods. **(A)** Frequency of the CD3^+^ population and number of CD3^+^CD4^+^ cells (Th cells) in WT and MR1^-/-^ mice. **(B)** Frequency and number of T-bet^+^ (Th1) cells (upper right panels) in the Th gate shown in **(A)**. **(C)** Representative flow cytometric profiles of CD3^+^CD4^+^ Foxp3^+^ and RORγt^+^ cell populations in WT or MR1^-/-^ mice. **(D)** Frequencies and numbers of RORγt^+^Foxp3^-^ (Th17; left panels), RORγt^-^Foxp3^+^ (Treg; middle panels), and RORγt^+^Foxp3^+^ (stable Treg effector; right panels) cells in WT and MR1^-/-^ mice represented by panel **(C)**. **(E)** Representative flow cytometric profiles of IL-17A or IFN-γ intracellular staining in CD3^+^CD4^+^ cells. Intracellular staining of IL-17A and IFN-γ in Th cells following stimulation with PMA and ionomycin for 4 h. T cells were obtained from draining lymph nodes of WT or MR1^-/-^ mice five days after sensitization at shaved abdominal skin sites. **(F)** Frequencies and cell numbers of IL-17A^+^ (left panels) or IFN-γ^+^ cell populations (right panels) represented by panel **(E)** Representative data of at least three experiments of three mice/experiment. Mann–Whitney *U* test. *p < 0.05.

Next, we examined the frequency and number of T helper (Th) subsets in the dLNs of WT and MR1^-/-^ mice after 5 days of sensitization. There were no differences in the frequencies or the numbers of CD4^+^CD3^+^ (T) cells in dLNs between WT and MR1^-/-^ mice (**Figure 5A**). When T-bet^+^ cells (Th1) were analyzed among CD4^+^CD3^+^ cells, the frequency of T-bet^+^ cells in the dLNs of MR^-/-^ mice was lower than that in WT mice, and the number of T-bet^+^ CD4^+^ T cells also exhibited a decreasing trend (**Figure 5B**). The CD4^+^ T cells were analyzed for RORγt and Foxp3 expression (**Figure 5C**). The frequencies and numbers of RORγt^+^Foxp3^-^ (Th17) cells were significantly higher in MR1**^-/-^** than in WT mice (**Figure 5D**, upper left and lower left panels). There were no differences in the frequency and number of RORγt^-^Foxp3^+^ (Treg) cells between WT and MR1^-/-^ mice (**Figure 5D**, upper and lower middle panels). Of note, RORγt^+^Foxp3^+^ cells, which may represent stable Treg effector cells (26), although a small population in comparison with RORγt^-^Foxp3^+^ cells, appeared more frequently (2×) in MR1^-/-^ mice than in WT mice, as shown in **Figure 5C**. However, there were no statistical differences in the mean frequencies and cell numbers of the population between WT and MR1^-/-^ mice (**Figure 5D**, upper and lower right panels). Additionally, there were no differences in each fraction of Th cells in unsensitized mice (**Supplementary Figures 4A, B**). Consistent with the above findings, staining for intracellular cytokines in CD4^+^ T cells treated *in vitro* with PMA and ionomycin for 4 h (**Figure 5E**) demonstrated that the CD4^+^ T cells of MR1**^-/-^** mice exhibited a higher frequency and number of IL-17A^+^ cells than those of WT mice (**Figure 5F**, upper and lower left panels), whereas frequency and number of IFN-γ^+^ cells differed between WT and MR1^-/-^ mice (**Figure 5F**, upper and lower right panels). These cytokine profiles are consistent with the data obtained from the culture experiments in **Figure 4**. Comparable production of IFN-γ protein was found in the culture supernatant detected by CBA (**Figure 4**), as intracellular protein detected by flow cytometry (**Figure 5E**), whereas T-bet^+^ T cells were reduced in frequency (**Figure 5B**).

### 3.5 Increased IL-17A-producing dermal γδ T cells in MR1^-/-^ mice

**Figure 6:**
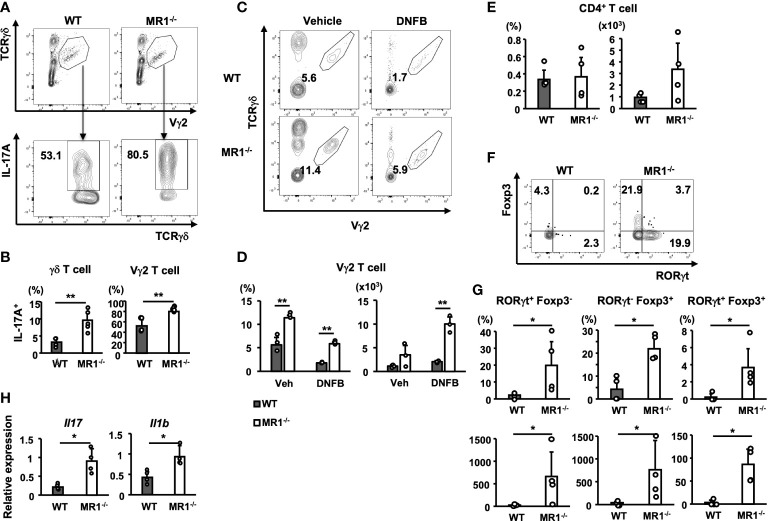
T-cell subsets in pinnae of unsensitized or sensitized mice and gene expression in sensitized pinnae. **(A)** Flow cytometric profiles of Vγ2^+^ γδ T cells (gated as the polygon; upper panels) in γδ T cells and intracellular IL-17A in gated Vγ2^+^ T cells (lower panels) in WT and MR1^-/-^ mice. T cells obtained from unsensitized pinnae were stimulated with PMA and ionomycin *in vitro* for 4 h. The expression of intracellular IL-17A was then analyzed in the Vγ2^+^ population in the total γδ T cells by flow cytometry. **(B)** Frequency of IL-17A^+^ population in the total γδ T cells (left panel) or in the Vγ2^+^ cells (right panel) in WT and MR1^-/-^ mice represented by panel **(A)**. **(C)** Representative flow cytometric profiles of dermal Vγ2^+^ γδ T cells (gated as the polygon) in vehicle control and DNFB-painted pinnae in WT and MR1^-/-^ mice two days after challenge. **(D)** Frequencies and numbers of Vγ2^+^ γδ T cells in vehicle-painted (veh) and DNFB-painted (DNFB) pinnae. **(E)** Frequencies and numbers of CD4^+^CD3^+^ cells (Th) in DNFB-painted pinnae of WT and MR1^-/-^ mice. **(F)** Flow cytometric profiles of RORγt and Foxp3 staining for the Th cells exhibited in E for WT (left panel) and MR1^-/-^ (right panel) mice. **(G)** Frequencies and numbers of RORγt^+^Foxp3^-^ (Th17; left panels) cells and RORγt^-^Foxp3^+^ (Treg; middle panels) cells represented in panel **(F)**. **(H)** Relative expression of *Il17* (left panel) and *Il1b* (right panel) mRNA in pinnae 6 h after DNFB challenge in WT and MR1^-/-^ mice. Representative data of at least two experiments of three to five mice/experiment. Mann–Whitney *U* test. *p < 0.05, **p < 0.01.

We then examined the population of T cells in the pinnae of MR1^-/-^ mice, because the source of IL-17A production was assumed to be Th17 cells as well as Tγδ17 cells (27). Notably, the pinnae of the vehicle control mice contained dermal T cells at a higher frequency in MR1^-/-^ mice (**Figures 3A, B**). Der γδ T cells, especially Vγ2^+^ γδ T cells, contain the Tγδ17 cell population in the skin (26). Thus, we examined Der γδ T cells for IL-17 expression in pinnae of unsensitized WT or MR1^-/-^ mice after stimulation with PMA and ionomycin *in vitro*. Not only total T cells but also Der Vγ2^+^ T cells exhibited a high frequency of IL-17A^+^-T cells in the pinnae of WT mice and an even higher frequency in MR1^-/-^ mice than in WT mice under unsensitized conditions (**Figures 6A, B**). When Vγ2^-^ Epi γδ and Der γδ T cells were analyzed for IL-17A in the same settings as in **Figure 6A** (gated as Vγ2^-^ for **Supplementary Figure 5A**), Vγ2^-^ Der γδ T cells in MR1^-/-^ mice were also significantly increased, but to a lesser extent than Vγ2^+^ Der γδ T cells, whereas the Vγ2^-^ Epi γδ T cells exhibited a decreasing trend (*p* = 0.05) in MR1^-/-^ mice compared to WT mice in terms of the frequency of IL-17A^+^-cells (**Supplementary Figures 5B, C**).

We next examined Der γδ T cells for the expression of Vγ2 in DNFB-challenged pinnae two days after elicitation (**Figure 6C**). The number of Der γδ T cells that expressed the Vγ2 chain increased, even with a decreasing trend for the infiltration of αβ T cells (**Figure 6D**).

To examine CD4^+^ T cells in DNFB-challenged pinnae, we analyzed the cells obtained on day 2 of elicitation, and a similar frequency was observed for WT and MR1^-/-^ mice, although an increasing trend in the number of Th cells was observed in MR1^-/-^ mice compared to WT mice (**Figure 6E**). The CD4^+^ T cells were also analyzed for the expression of Foxp3 and RORγt (**Figure 6F**). Both the frequency and cell number of all subsets, RORγt^+^Foxp3^-^ (Th17; **Figure 6G**, left panels), RORγt^-^Foxp3^+^ (Treg; **Figure 6G**, middle panels), and RORγt^+^Foxp3^+^ (stable Treg effector; **Figure 6G**, right panels), were increased in MR1^-/-^ mice compared with those in WT mice. To explain the upstream events that led to the above differences, the relevant cytokine mRNAs were analyzed 6 h after elicitation. Both *Il17* and *Il1b* expression were significantly increased in the pinnae of MR1^-/-^ mice compared with WT mice as early as 6 h (**Figure 6H**), suggesting that the expression of IL-1β might enhance the responses of both Th17 and Tγδ17 cells. The ear swelling induced by DNFB challenge in MR1^-/-^ mice was already augmented at 6 h (**Supplementary Figure 6A**), and the expression of genes relevant to neutrophils, such as *Csf3*, *Cxcl1*, and *Cxcl2*, was also increased (**Supplementary Figure 6B**), although neutrophil recruitment was comparable at this time point between WT and MR1^-/-^ mice (**Supplementary Figure 6C**).

## 4 Discussion

In the present study, we demonstrated that ACD was augmented in MR1^-/-^ mice compared to WT mice because of the increased numbers of Th17 and Tγδ17 cells in MR1^-/-^ mice. MAIT cells were markedly reduced upon elicitation with DNFB in WT mice. MAIT cells (5-OP-RU/MR1 tetramer^+^ cells) in the dLN on day 3 of DNFB challenge expressed Nur77 in Nur77*^gfp^* mice (data not shown), suggesting that MAIT cells were activated during the elicitation phase. Furthermore, the deficiency of MAIT cells appears to be related to an altered distribution and/or number of T cells and a bias towards the type 3 immune response in a direct or indirect manner, although the mechanism remains elusive.

MR1 deficiency may cause wider defects in MR1-restricted T cells (MR1T) (28) besides MAIT cells, as the diversity of MR1T (including MAIT and MR1-reactive T) cells extends to six different groups with unique modes of recognition, binding, and reactivity (29), most of which are αβ type but include a γδ type, such as Vδ3Vγ8 T cells that bind and recognize MR1 at its membrane proximal region, similar to an α3 domain-recognizing antibody (30, 31). Notably, the significant role of MR1T cells have been shown to play an important role in antitumor immunity (32) and have already been implicated in infectious and autoimmune diseases (33). It should be noted that T cells obtained from Vα19Jα33^Tg^Cα^-/-^ mice that overexpress MAIT cells were previously tested for suppression of delayed-type hypersensitivity (34). Transfer of invariant Vα19^+^ T cells but not control non-transgenic T cells suppressed foot pad swelling induced by sheep red blood cells in B6 hosts prior to sensitization, accompanied by a reduction of serum IL-17 and IFN-γ. Nevertheless, further studies will be needed to explore the mechanisms by which MR1T/MAIT cells interact with other immune cells to suppress ACD response.

The significant increase and bias towards Tγδ17 cells we observed in the skin of MR1^-/-^ mice is likely associated with the enhanced ACD response, although the timing and site of the developmental characteristics (35) in the biased distribution of Vγ2^+^ Tγδ17 in MR1^-/-^ mice remain to be determined. Interestingly, MAIT cells and γδ T cells have a reciprocal relationship, similar to the expansion of MAIT cells in NKT cell-deficient mice (36). For instance, a patient with a homologous MR1 mutation at position 31 Arg to His substitution (position 9 in mature MR1 protein: MR1^R9H^/MR1^R9H^) was discovered to display primary immunodeficiency due to functional MR1 deficiency, with no circulating MAIT cells (37). Notably, the patient had increased circulating T cells expressing Vγ9Vδ2 with the CD27^-^CD28^-^ phenotype. Conversely, MAIT cells have been reported to be increased in γδ^-/-^ mice (38). These results indicate that there is an equipoise among the three types (NKT, MAIT, and γδ) of innate-like T cells by competing with a homeostatic factor or niche (39). Hence, it is tempting to speculate that NKT cells, MAIT cells and γδ T cells all contribute in a reciprocal manner, as a ménage à trois, to various inflammatory diseases such as ACD. The present study demonstrated that in MR1^-/-^ mice, the dominant Vγ2^+^ Tγδ17 cells in the skin (**Figure 6**) and increased Th17 cells (**Figure 5**) upon sensitization in the dLN migrated to the skin and enhanced ACD. It is intriguing that skin MAIT cells are biased towards IL-17 production (MAIT17) and promote tissue repair (38), and that their deficiency appears to be compensated by the dominance of Tγδ17 cells in the skin. It is not known whether there are any direct interactions between MAIT cells and γδ T cells that limit each other’s effector functions. However, one may speculate that MAIT cells and γδ T cells compete with each other for homing niches within the dermis, where MAIT cells localize near the dermal-epidermal interface (38) and γδ T cells localize to also in superficial regions (40). The most critical factor for MAIT cell tissue homing and homeostasis is likely their early life exposure to and sustained interaction with the microbiota that synthesize riboflavin (38). Tγδ17 cells are similarly influenced by microbes for their expansion and functional activity (41), suggesting that skin commensals affects the balance between MAIT cells and γδ T cells. Furthermore, cytokines such as IL-1β and IL23 (38) in the environment are also thought to be important factors that affect the balance between these T cell subsets.

The macroscopic and microscopic appearances of skin pathology were markedly enhanced with edema and cellular infiltration in MR1^-/-^ mice compared with WT mice (**Figure 1**). In severe cases in MR1^-/-^ mice, the elicited pinnae were covered with crustae by frequent scratching, and a large area of the inflammatory lesion was sometimes lost, presumably due to necrosis or injury, which was not observed in WT mice. Thus, the skin thickness data for severe cases were inevitably unincorporated in the analyses. The severity of dermatitis may permit use of ACD in MR1^-/-^ mice as an intractable model system to study disease pathogenesis and testing immune therapies. Notably, MAIT cells have been reported to display tissue repair functions, as wound healing by punch biopsy was significantly delayed in the absence of MAIT cells (38). If the keratinocytes injured during ACD by cytotoxic lymphocytes fail to be replaced with newly proliferated cells, the epithelial defect may cause infections and further damage the skin. A recent study also revealed that amphiregulin, a member of the epidermal growth factor family produced by MAIT cells, accelerated wound closure, but in an MR1-independent manner (42). In experimental autoimmune uveoretinitis, MAIT cells ameliorated disease, which was associated with anti-inflammatory/neuroprotective activities of IL-22 as well as IL-22-independent repair functions upon stimulation with 5-OP-RU (43). Accordingly, the severity of ACD response in MR1^-/-^ mice observed in our study may result in part from defective repair due to MR1T/MAIT cell deficiency.

In the absence of exogenous stimulation, MR1^-/-^ mice exhibited a similar pinna thickness compared to WT mice (**Figure 1C**), suggesting that MR1^-/-^ mice do not develop spontaneous dermatitis. However, increased production of IL-1β in mutant mice than WT mice was detected at pinnae after 6 h of elicitation with DNFB (**Figure 6H**), since the barrier function of the skin was presumably weakened in MR1^-/-^ mice due to MAIT cell deficiency (38, 44). The ear swelling in MR1^-/-^ mice was more enhanced than WT mice at 6 h of elicitation (**Supplementary Figure 6A**), whereas the level of neutrophil migration was similar between the two strains (**Supplementary Figure 6C**), suggesting that edematous changes at the very early phase appeared to be different between MR1^-/-^ and WT mice.

The cellular infiltrates consisted mainly of Mo/Mϕ, neutrophils, and αβ T cells in both MR1^-/-^ and WT mice after DNFB elicitation (**Figure 2** and **Supplementary Figures 2**, **3**). Notably, there were significantly more neutrophils in terms of percentage and actual cell numbers in MR1^-/-^ mice than WT mice. The recruitment of infiltrates was concordant with the enhanced expression of cytokines and chemokines by Th17 and Tγδ17 cells in the pinnae stained with DNFB, which supported neutrophil generation, recruitment, and activation (**Figures 2C**, **4**). Resident Mϕ were reduced in percentage due to, in part, dilution by the recruitment of Mo/Mϕ and a reduction in the actual cell number in both WT and MR1^-/-^ mice (**Supplementary Figures 2**, **3**). As for eosinophils in pinnae, the cell number per 10 mg tissue was not significantly increased in DNFB-challenged pinnae in MR1^-/-^ mice compared with WT mice (data not shown). Although these changes result from the MR1T/MAIT cell deficiency, the underlying mechanisms remain to be further investigated.

When the T cells were compared in MR1^-/-^ and WT mice, a large number of αβ-type T cells specific for the sensitizer Ag in pinnae was equally recruited in both MR1^-/-^ and WT mice after challenge with DNFB. Thus, the percentage of Epi and Der γδ T-cell fractions decreased accordingly after challenge (**Figure 3B**). The apparent reduction was simply due to dilution by the migrated αβ T cells into the pinnae, whereas the number of γδ T cells in each fraction increased after challenge to enhance the ACD response *via* production of cytokines and chemokines from Th17 and Tγδ17 cells. Notably, MR1^-/-^ mice harbored a significantly higher percentage of Der γδ T cells, even in unsensitized states, and exhibited a higher percentage of IL-17A^+^ cells in both the Vγ2^+^ and Vγ2^-^ fraction (Vγ2^+^ > Vγ2^-^) upon *in vitro* stimulation with PMA and ionomycin (**Figures 6C, D**; **Supplementary Figures 5B, C**). The abundance of Tγδ17 cells in the skin of MR1^-/-^ mice may result in a robust type 3 immune response at the site of ACD since more Tγδ17 cells during the initiation phase in the dermis effectively boosted the response compared with WT mice.

The frequency of MAIT cells in mouse skin is strikingly different from that in human skin, with approximately 10% of αβ T cells being MAIT cells in mice and 0.5%-2% of αβ T cells being MAIT cells in humans, with the remainder being the conventional type and NKT cells (38, 44). Therefore, the present results must be considered when assessing whether they are readily applicable to human cases of ACD. However, the involvement of innate T cells in ACD is not compromised in humans, as iNKT cells presumably participate as effectors (21) and the role of NKT cells in ACD may vary depending on different sensitizers (19). MAIT cells were detected in palladium allergy in a previous report (22), and the involvement of iNKT cells has already been demonstrated in allergies to metals such as nickel, cobalt, and chromium (21, 45–47) that are present in accessories, biomedical devices, and food constituents (1). It is intriguing to consider whether MAIT cells and iNKT cells adopt a common or distinct pathway that affects the ACD response. Notably, MAIT cells have been examined as promising targets for immunotherapy in the skin for phototherapy of atopic dermatitis (48) and as effectors of a major inflammatory disease, psoriasis (13). The utilization of MAIT ligands as therapeutic agents may be associated with low resistance by patients, since they are vitamin B-related compounds with either inhibitory (VB_9_ -folate) or stimulatory (VB_2_ -riboflavin) activities (14, 49). To examine whether MAIT cells can be modulated to protect against ACD, further investigations that clarify their immunoregulatory role will be required.

## Data availability statement

The original contributions presented in the study are included in the article/**Supplementary Materials**. Further inquiries can be directed to the corresponding author.

## Ethics statement

The animal study was reviewed and approved by the Animal Experimentation and Ethics Committee of Kitasato University School of Medicine (#2017-143, 2018-119, 2019-025, and 2022-079).

## Author contributions

NI, MS and KI designed the studies and wrote the manuscript. NI, MS, EC and KY performed and analyzed the experiments. MG and KI supervised the work. All authors contributed to the article and approved the submitted version.

## Supplementary material

Supplementary Figure 1 Gating strategy for the flow cytometric analyses performed in the present study. A. Gating strategy for detecting granulocytes. The acquisition was mostly ungated except for small particles (with very low FSC-A) and the acquired cells were widely gated with FSC-A/SSC-A as shown to include larger cells with intracellular granules. Cells were removed from doublets and dead cells followed by gating for the CD45^+^ population as shown in sequence. Finally, CD11b^+^Ly6G^+^ cells in the CD45^+^ cell population were designated as neutrophils. B. Gating strategy for detecting T-cell subsets. The acquired cells were lymphocyte-gated in tighter FSC-A/SSC-A than that of A, as shown. The CD45^+^ population after removal of doublets and dead cells was further analyzed with TCRβ/TCRγδ or TCRγδ/Vγ2 to discriminate αβ T, γδ T, and Vγ2^+^ T subsets in the γδ T cell population.

Supplementary Figure 2 Monocytes (Mo) and macrophages (Mϕ) in the ACD-induced pinnae in WT and MR1^-/-^ mice. Cells infiltrated into the pinna were prepared two days after challenge with enzymatic degradation as described in the *Materials and Methods* and analyzed by flow cytometry as described for **Supplementary Figure 1A**. Flow cytometric profiles of inflammatory cells in the vehicle- and DNFB-painted pinnae in WT and MR1**^-/-^** mice, as shown in . The cells of the CD11b^+^Ly-6G^lo-(-)^ population were further separated into Ly-6C^hi^F4/80^lo^ (Mo/Mϕ) and Ly-6C^-^F4/80^hi^ (Mϕ). B. Frequency of Mo/Mϕ (upper panel) or Mϕ (lower panel) in vehicle- and DNFB-painted pinnae of WT and MR1^-^ **^/-^** mice at day 2 after elicitation represented by panel A. Representative data of at least three experiments of three mice/experiment.

Supplementary Figure 3 Cellular composition of mononuclear cells obtained from vehicle- and DNFB-painted pinnae of WT and MR1^-/-^ mice. Each fraction of cells was recapitulated from the results of flow cytometric analyses according to the gating described for **Supplementary Figure 1**. Neu: neutrophil (CD11b^+^Ly-6G^hi^); Mϕ: macrophage (CD11b^+^Ly-6G^lo-(-)^Ly-6C^lo^/F4/80^hi^); Mo/Mϕ: monocyte/macrophage lineage (CD11b^+^Ly-6C^lo-(-)^Ly-6C^hi^/F4/80^lo^); αβ: αβ T cells (CD3^+^TCRβ^+^TCRγ/δ^-^), γδ^hi^: epidermal γδ T cells (CD3^+^TCRβ^-^TCRγ/δ^hi^); and γδ^lo^: dermal γδ T cells (CD3^+^TCRβ^-^TCRγ/δ^lo^). Representative data of at least three experiments of three mice/experiment.

Supplementary Figure 4 T-helper (Th) cell subsets in draining lymph nodes from unsensitized WT and MR1^-/-^ mice. Cells in inguinal lymph nodes were obtained from each unsensitized strain of mice and stained for the analyses according to the *Materials and Methods*. **A**. Representative flow cytometric profiles of CD3^+^CD4^+^ cells of the Foxp3^+^ and RORγt^+^ population in WT and MR1^-/-^ mice. **B**. Frequencies and cell numbers of RORγt^+^Foxp3^-^ (Th17; left panels), RORγt^-^Foxp3^+^ (Treg; middle panels), and RORγt^+^Foxp3^+^ (stable Treg effector; right panels) cells in WT and MR1^-/-^ mice represented in panel A. Representative data of at least two experiments of four mice/experiment.

Supplementary Figure 5 IL-17A expression in epidermal γδ T cells and Vγ2^-^ dermal γδ T cells in unsensitized mice. **A**. Gating of Vγ2^-^ epidermal and dermal γδ T cells in flow panels of WT and MR1^-/-^ mice. T cells obtained from unsensitized pinnae were stimulated with PMA and ionomycin *in vitro* for 4 h. **B**. The expression of intracellular IL-17A was analyzed in the Vγ2^-^ population in epidermal and dermal γδ T cells by flow cytometry. **C**. Frequency of the IL-17A^+^ population in Vγ2^-^ epidermal (upper panel) and dermal γδ T cells (lower panel) in WT and MR1^-/-^ mice represented in panel B. Representative data of at least three experiments of four mice/experiment. Mann–Whitney *U* test. **p* < 0.05.

Supplementary Figure 6 MR1^-/-^ mice develop an augmented response at 6 h after DNFB challenge. **A**. WT (closed circle) and MR1^-/-^ (open circle) mice were sensitized and challenged on the left pinna with vehicle only or on the right pinna with DNFB. The thickness of the pinnae was then measured with a digital micrometer 6 h after challenge. The increment in thickness of the sensitized pinna represented as ΔEar swelling in **Figure 1A**. **B**. The expression of *Cxcl1*, *Cxcl2*, and *Csf3* related with neutrophil recruitment and activation was examined with mRNA obtained from the left pinnae (vehicle control) and the right pinnae (DNFB) of either WT (closed bar) or MR1^-/-^ mice (open bar) at 6 h after challenge. **C**. Frequency of neutrophils in the CD45^+^ fraction in MR1^-^ **^/-^** mice compared with those of WT mice as in 6 h after challenge (vehicle: closed bar, DNFB: open bar). Representative data of at least two experiments of four mice/experiment. Mann–Whitney *U* test. **p* < 0.05
