# Supplementary figures and images for: MR1 deficiency enhances IL-17-mediated allergic contact dermatitis
Source: Front Immunol. 2023 Jun 20;14:1215478. doi: 10.3389/fimmu.2023.1215478 (PMC10319069; doi:10.3389/fimmu.2023.1215478)

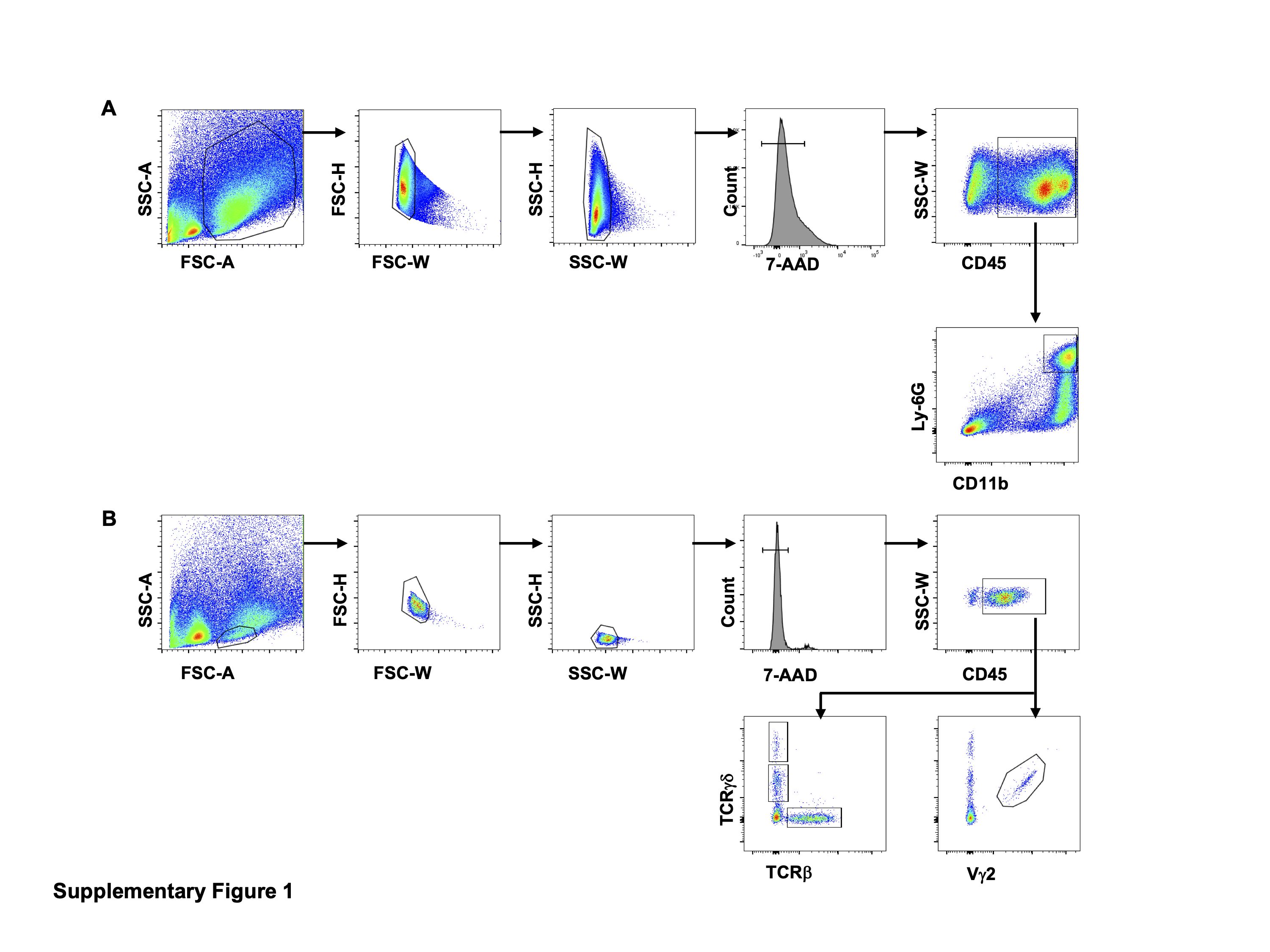

Supplement: Supplementary Figure 1 — Gating strategy for the flow cytometric analyses performed in the present study. A. Gating strategy for detecting granulocytes. The acquisition was mostly ungated except for small particles (with very low FSC-A) and the acquired cells were widely gated with FSC-A/SSC-A as shown to include larger cells with intracellular granules. Cells were removed from doublets and dead cells followed by gating for the CD45+ population as shown in sequence. Finally, CD11b+Ly6G+ cells in the CD45+ cell population were designated as neutrophils. B. Gating strategy for detecting T-cell subsets. The acquired cells were lymphocyte-gated in tighter FSC-A/SSC-A than that of A, as shown. The CD45+ population after removal of doublets and dead cells was further analyzed with TCRβ/TCRγδ or TCRγδ/Vγ2 to discriminate αβ T, γδ T, and Vγ2+ T subsets in the γδ T cell population. [file Image_1.jpg]

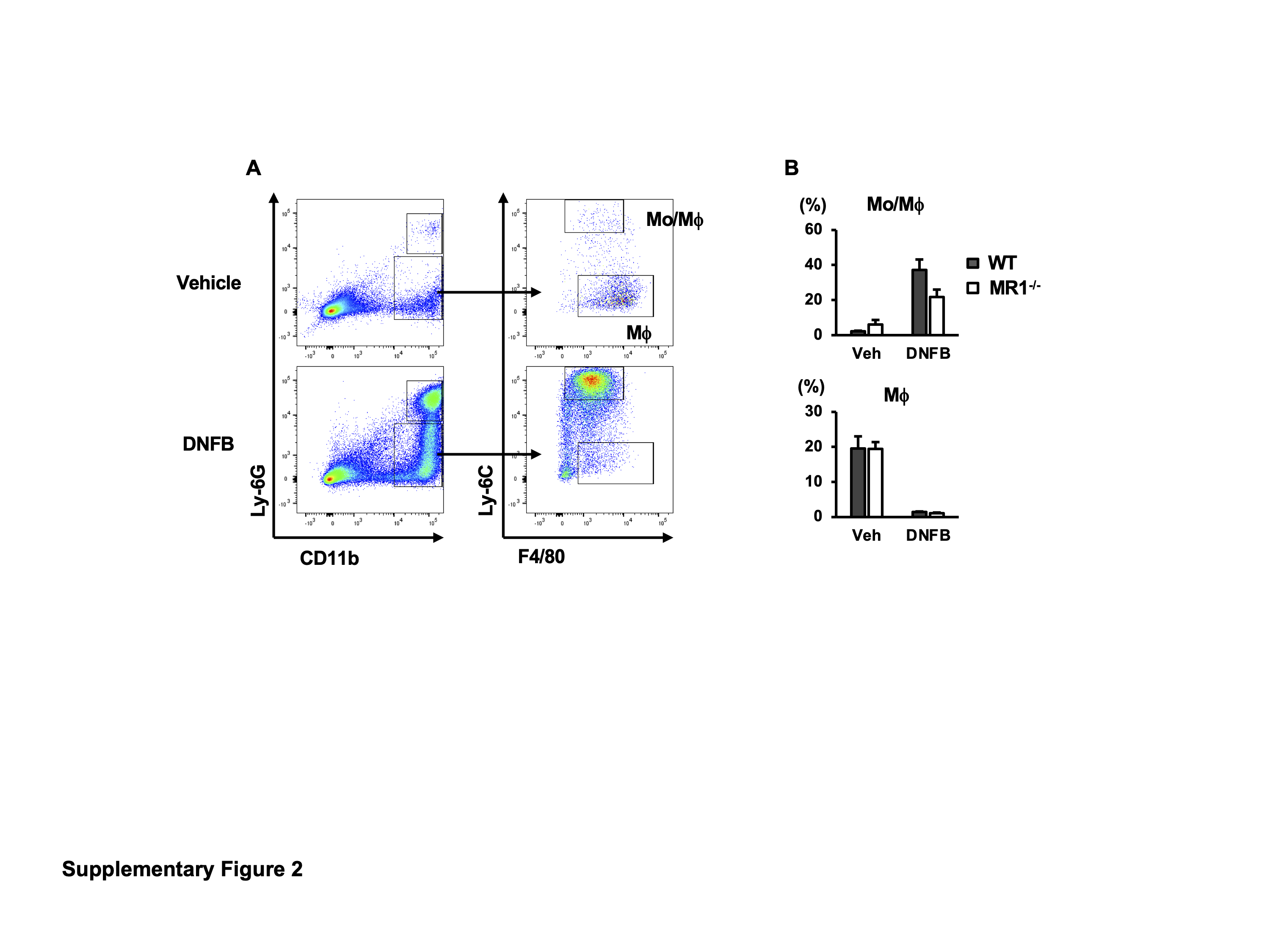

Supplement: Supplementary Figure 2 — Monocytes (Mo) and macrophages (Mϕ) in the ACD-induced pinnae in WT and MR1-/- mice. Cells infiltrated into the pinna were prepared two days after challenge with enzymatic degradation as described in the Materials and Methods and analyzed by flow cytometry as described for Supplementary Figure 1A . Flow cytometric profiles of inflammatory cells in the vehicle- and DNFB-painted pinnae in WT and MR1 -/- mice, as shown in . The cells of the CD11b+Ly-6Glo-(-) population were further separated into Ly-6ChiF4/80lo (Mo/Mϕ) and Ly-6C-F4/80hi (Mϕ). B. Frequency of Mo/Mϕ (upper panel) or Mϕ (lower panel) in vehicle- and DNFB-painted pinnae of WT and MR1- /- mice at day 2 after elicitation represented by panel A. Representative data of at least three experiments of three mice/experiment. [file Image_2.jpg]

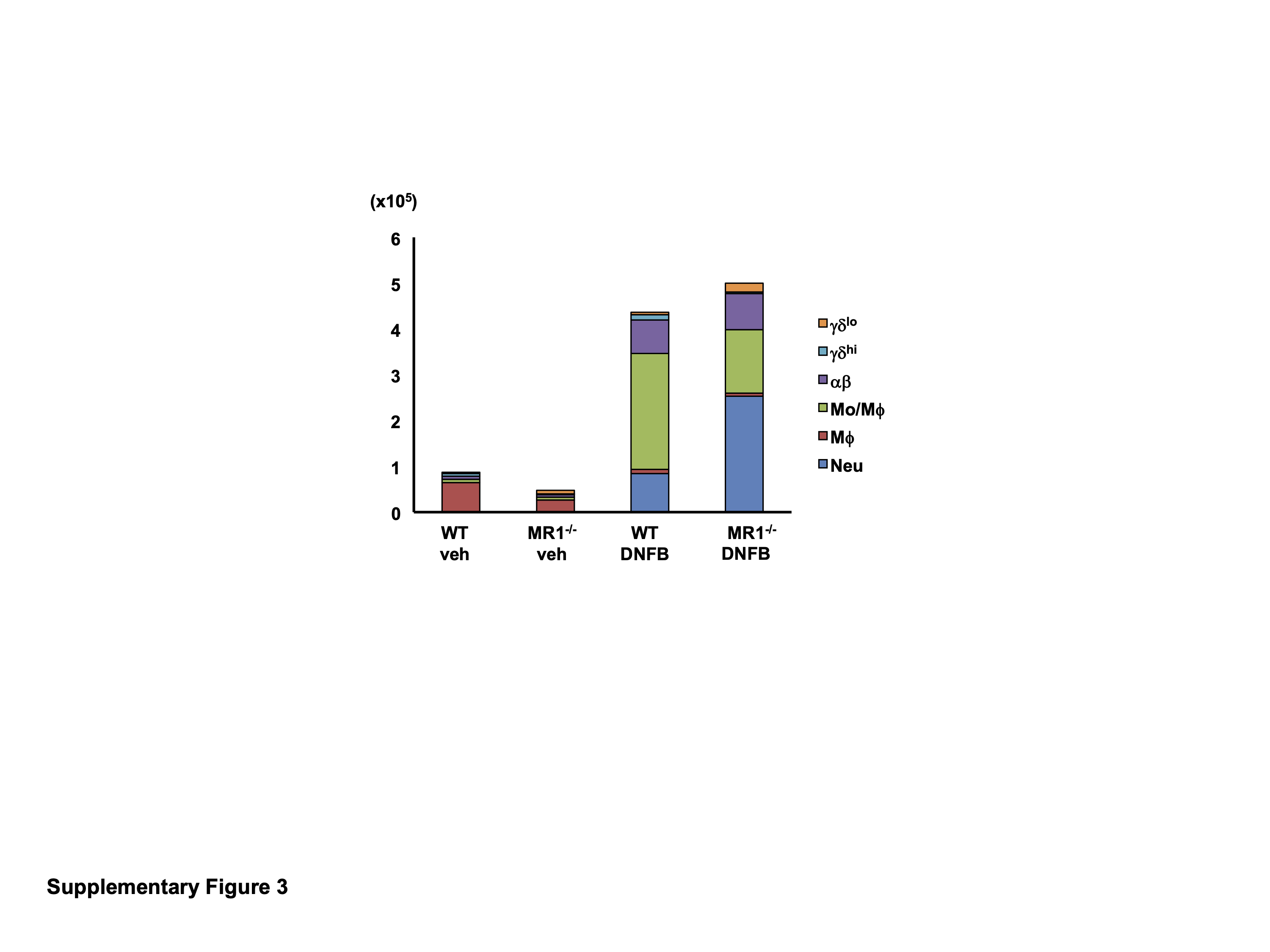

Supplement: Supplementary Figure 3 — Cellular composition of mononuclear cells obtained from vehicle- and DNFB-painted pinnae of WT and MR1-/- mice. Each fraction of cells was recapitulated from the results of flow cytometric analyses according to the gating described for Supplementary Figure 1 . Neu: neutrophil (CD11b+Ly-6Ghi); Mϕ: macrophage (CD11b+Ly-6Glo-(-)Ly-6Clo/F4/80hi); Mo/Mϕ: monocyte/macrophage lineage (CD11b+Ly-6Clo-(-)Ly-6Chi/F4/80lo); αβ: αβ T cells (CD3+TCRβ+TCRγ/δ-), γδhi: epidermal γδ T cells (CD3+TCRβ-TCRγ/δhi); and γδlo: dermal γδ T cells (CD3+TCRβ-TCRγ/δlo). Representative data of at least three experiments of three mice/experiment. [file Image_3.jpg]

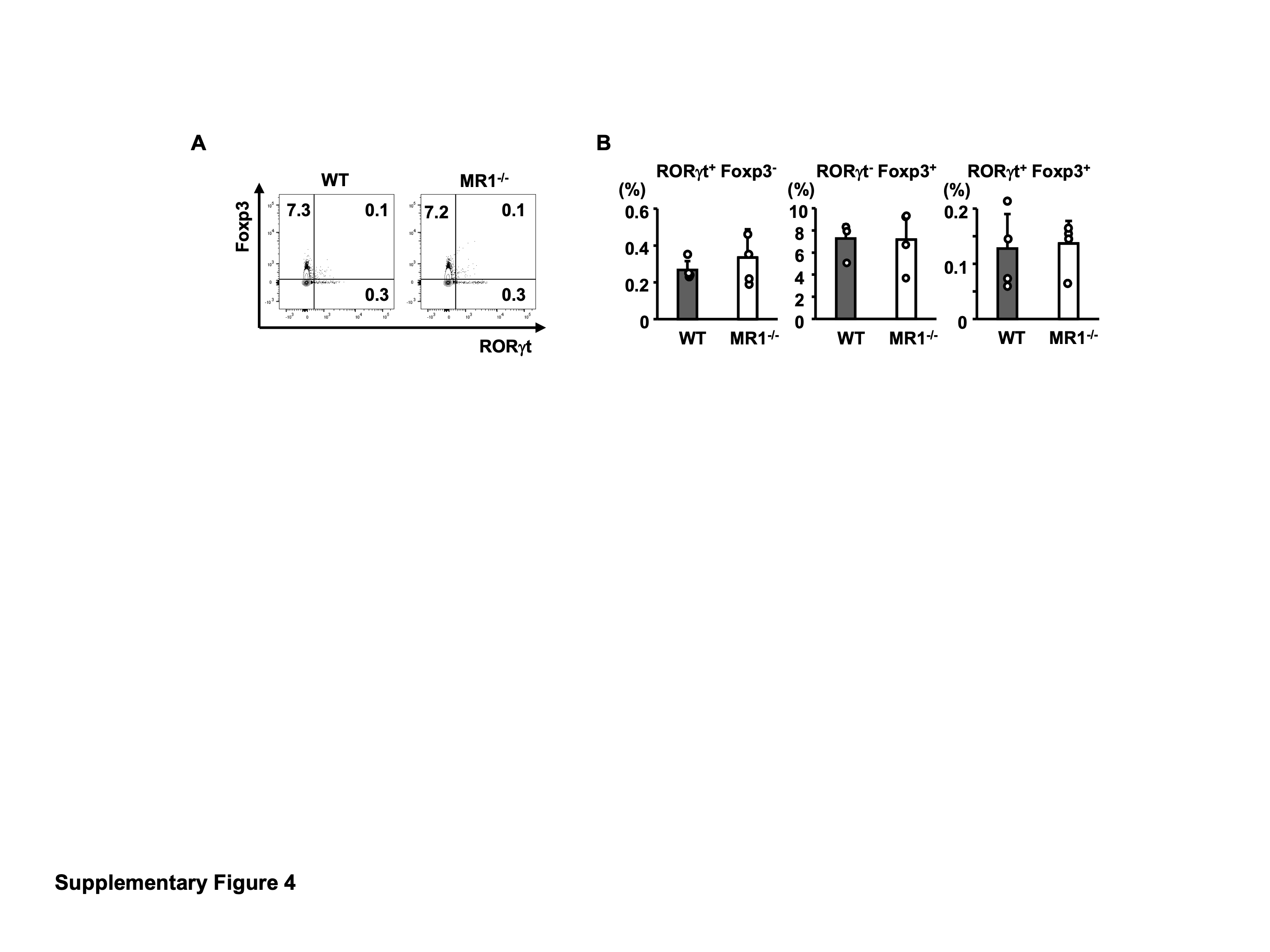

Supplement: Supplementary Figure 4 — T-helper (Th) cell subsets in draining lymph nodes from unsensitized WT and MR1-/- mice. Cells in inguinal lymph nodes were obtained from each unsensitized strain of mice and stained for the analyses according to the Materials and Methods. A. Representative flow cytometric profiles of CD3+CD4+ cells of the Foxp3+ and RORγt+ population in WT and MR1-/- mice. B. Frequencies and cell numbers of RORγt+Foxp3- (Th17; left panels), RORγt-Foxp3+ (Treg; middle panels), and RORγt+Foxp3+ (stable Treg effector; right panels) cells in WT and MR1-/- mice represented in panel A. Representative data of at least two experiments of four mice/experiment. [file Image_4.jpg]

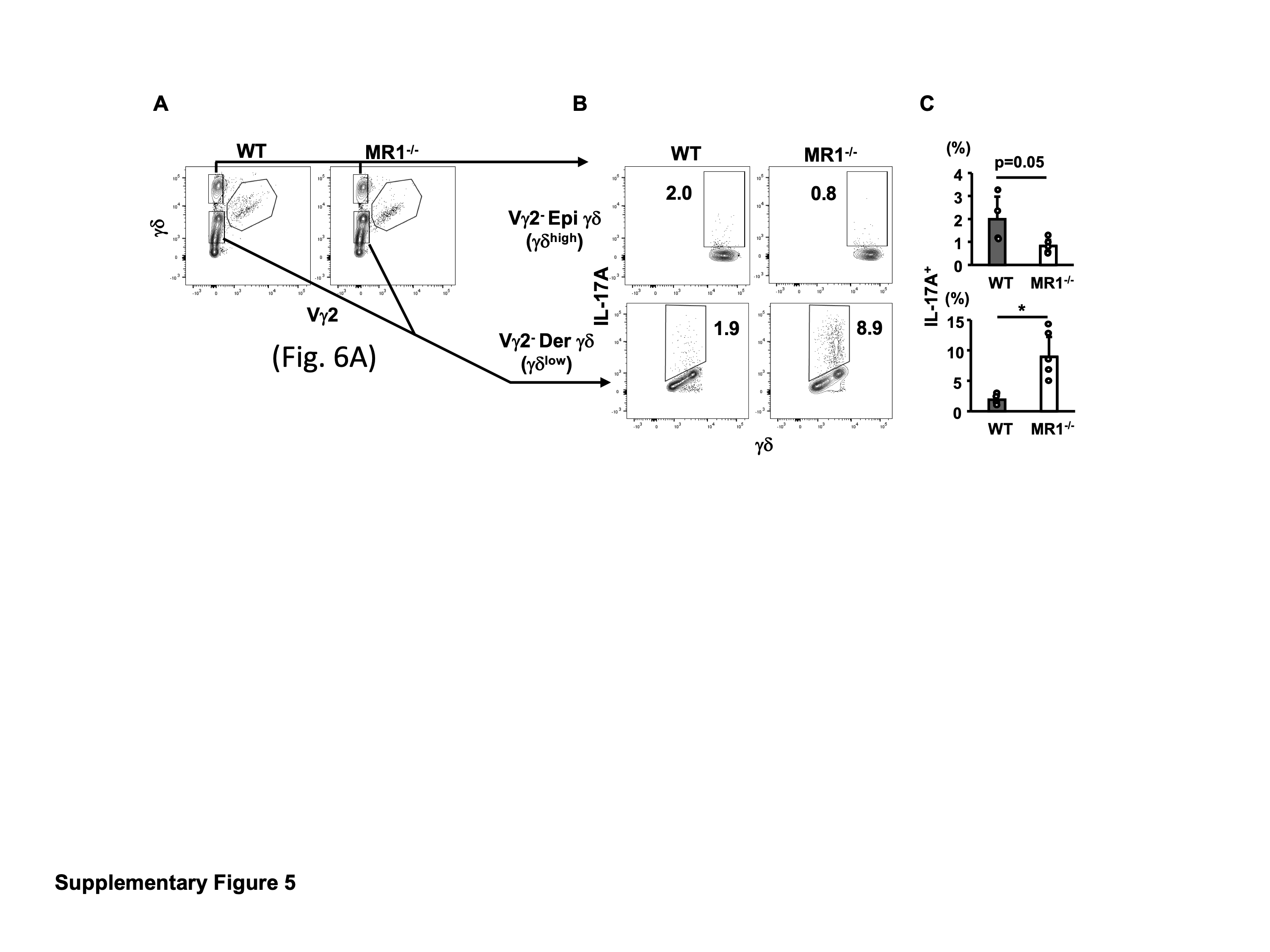

Supplement: Supplementary Figure 5 — IL-17A expression in epidermal γδ T cells and Vγ2- dermal γδ T cells in unsensitized mice. A. Gating of Vγ2- epidermal and dermal γδ T cells in flow panels of WT and MR1-/- mice. T cells obtained from unsensitized pinnae were stimulated with PMA and ionomycin in vitro for 4 h. B. The expression of intracellular IL-17A was analyzed in the Vγ2- population in epidermal and dermal γδ T cells by flow cytometry. C. Frequency of the IL-17A+ population in Vγ2- epidermal (upper panel) and dermal γδ T cells (lower panel) in WT and MR1-/- mice represented in panel B. Representative data of at least three experiments of four mice/experiment. Mann–Whitney U test. *p < 0.05. [file Image_5.jpg]

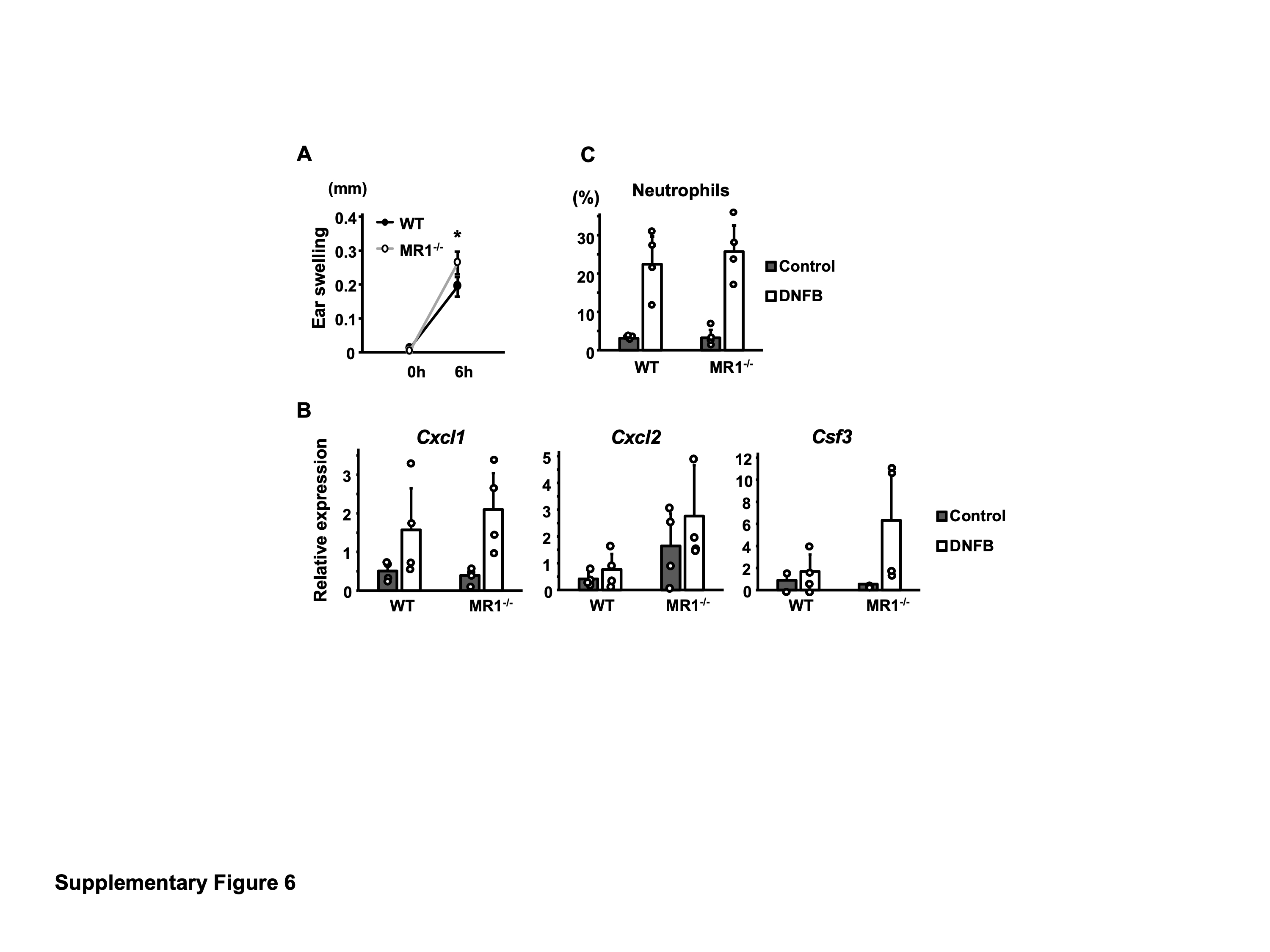

Supplement: Supplementary Figure 6 — MR1-/- mice develop an augmented response at 6 h after DNFB challenge. A. WT (closed circle) and MR1-/- (open circle) mice were sensitized and challenged on the left pinna with vehicle only or on the right pinna with DNFB. The thickness of the pinnae was then measured with a digital micrometer 6 h after challenge. The increment in thickness of the sensitized pinna represented as ΔEar swelling in Figure 1A. B. The expression of Cxcl1, Cxcl2, and Csf3 related with neutrophil recruitment and activation was examined with mRNA obtained from the left pinnae (vehicle control) and the right pinnae (DNFB) of either WT (closed bar) or MR1-/- mice (open bar) at 6 h after challenge. C. Frequency of neutrophils in the CD45+ fraction in MR1- /- mice compared with those of WT mice as in 6 h after challenge (vehicle: closed bar, DNFB: open bar). Representative data of at least two experiments of four mice/experiment. Mann–Whitney U test. *p < 0.05 [file Image_6.jpg]
